# Supplementary material for: Collaborative intelligence in AI: Evaluating the performance of a council of AIs on the USMLE
Source: PLOS Digit Health. 2025 Oct 9;4(10):e0000787. doi: 10.1371/journal.pdig.0000787 (PMC12510544; doi:10.1371/journal.pdig.0000787)
Supplement: S2 File — (ZIP) [file pdig.0000787.s002.zip › S2 Data Raw data files containing unprocessed question inputs and LLM outputs including transcripts of deliberations/STEP3_Question-70-1_Type-MC-NJ.docx]

**Facilitator** **[Round 1]:** A new diagnostic test for HIV infection is developed. The new serum biomarker concentration is measured in 711 men and women, ages 25 to 65 years. Of these individuals, 336 were previously determined to have HIV and 375 do not have HIV. Stratified results of the new serum biomarker test are shown: Serum Biomarker Concentration >100 U/mL Positive for HIV 120 Negative for HIV 10 Serum Biomarker Concentration 76–100 U/mL Positive for HIV 100 Negative for HIV 130 Serum Biomarker Concentration 51–75 U/mL Positive for HIV 75 Negative for HIV 75 Serum Biomarker Concentration 26–50 U/mL Positive for HIV 30 Negative for HIV 60 Serum Biomarker Concentration 0–25 U/mL Positive for HIV 11 Negative for HIV 200 Total Number of Positive for HIV = 336 Total Number of Negative for HIV = 375 Which of the following is the likelihood ratio that is associated with a serum biomarker concentration greater than 100 U/mL? (A) 2 (B) 8 (C) 13 (D) 25 (E) 33

**AI_E:** RESTATING THE QUESTION: The question is asking about the likelihood ratio attached to a serum biomarker concentration above 100 U/mL in a new HIV diagnostic test. The likelihood ratio is a statistical term used in diagnostic testing, which compares the probability of obtaining the test result in patients with the disease versus those without the disease. PLANNING: Assumptions include that the data provided is accurate, and the diagnostic test is reliable. The likelihood ratio for the test can be calculated from the given data. If the likelihood ratio is high, then the serum biomarker test is good at ruling in people with HIV when the serum concentration is greater than 100 U/mL. Going deeper, the likelihood ratio is the division of the proportion of patients with disease who test positive (true positive rate or sensitivity) over the proportion of patients without disease who test positive (false positive rate or 1-specificity). CLINICAL REASONING: From the data given, we can calculate the sensitivity and specificity at this cut-off point. Sensitivity is the ability of a test to correctly identify people who have a certain disease. In this case, it's the percentage of HIV-positive patients with a serum biomarker concentration >100 U/mL. Specificity, on the other hand, is a test's ability to correctly identify people who do not have a certain disease. In this case, it's the percentage of HIV-negative patients with a serum biomarker concentration ≤100 U/mL. CONCLUDING: We can calculate these values as follows: Sensitivity = [Number of true positives/(Number of true positives + Number of false negatives)] = [120/(120+216)] = 0.35714~ Specificity = [Number of true negatives/(Number of true negatives + Number of false positives)] = [365/(365+10)] = 0.97333~ So, the likelihood ratio would be Sensitivity/(1-Specificity) = 0.35714/(1-0.97333) ≈ 13. RESPONSE: Thus, the likelihood ratio that is associated with a serum biomarker concentration greater than 100 U/mL is (C) 13.

**AI_D:** RESTATING THE QUESTION: The question asks us to determine the likelihood ratio of a new diagnostic test for HIV based on the serum biomarker concentration greater than 100 U/mL. A likelihood ratio is a statistical measure used in epidemiology to compare the probability of a particular result in patients with a disease to the probability of the same result occurring in patients without the disease. This will help determine the test’s value in diagnosing the disease. PLANNING: The key aspects involved in calculating likelihood ratio include the true positive rate (sensitivity) and the false positive rate (1-specificity). To calculate the likelihood ratio for a positive test, we use the formula sensitivity/(1-specificity). Assuming that having a serum biomarker concentration greater than 100 U/mL is positive, we need to find the sensitivity and specificity of the test. CLINICAL REASONING: The sensitivity of a test is the ability to correctly identify individuals with a disease. In this context, it would be the ability of the test to correctly identify patients with HIV having a serum biomarker concentration greater than 100 U/mL. This would be the number of true HIV positive cases (120) divided by the total number of individuals with HIV (336). Specificity, on the other hand, is the ability of the test to correctly identify individuals without a disease. Here, it would be the ability of the test to correctly identify patients without HIV having a serum biomarker concentration less than or equal to 100 U/mL. This would be calculated as the number of true HIV negatives (365) divided by the total number of individuals without HIV (375). After calculating both the sensitivity and specificity, we can plug these values into the formula to get the likelihood ratio for the test at a biomarker concentration greater than 100 U/mL. CONCLUDING: Using the figures provided, the sensitivity is 120/336 (approximately 0.357 or 35.7%) and the specificity is 365/375 (approximately 0.973 or 97.3%). Plugging these values into the formula will give us the likelihood ratio. RESPONSE: With the values put into the formula, the likelihood ratio for a positive test is calculated to be roughly 13 (0.357/0.027). Therefore, the correct answer is (C) 13.

**AI_A:** RESTATING THE QUESTION: You're asking about the likelihood ratio of an innovative diagnostic test for HIV infection based on a serum biomarker concentration. This test has been implemented in a group of 711 individuals varying in age from 25 to 65 years. 120 individuals tested positive for HIV, and 10 tested negative when the serum biomarker concentration exceeded 100 U/mL. I have to figure out what the likelihood ratio for a serum biomarker concentration higher than 100 U/mL is. In medical diagnostic testing, the likelihood ratio is a measure of how much a test result changes the odds of having a disease, thus aiding in diagnosing disease. PLANNING: To answer the question, I need to calculate the likelihood ratio for a biomarker concentration >100 U/mL. This requires using the formula for Likelihood Ratio Positive (LR+), which equals the sensitivity of the test divided by (1 - specificity). Assumptions we're making: sensitivity is the proportion of individuals with HIV who are correctly identified by the test (>100 U/mL) and specificity is the proportion of individuals without HIV correctly identified by the test (<=100 U/mL). CLINICAL REASONING: The specificity and sensitivity values can be calculated using the provided test results. For sensitivity, out of the total number of patients diagnosed positive (336), 120 had a biomarker level of >100. So, Sensitivity = true positive/(true positive + false negative) = 120/ 336 For specificity, out of the total number of patients diagnosed as negative (375), 365 had a biomarker level ≤ 100 U/mL. Specificity = true negative/(true negative + false positive) = 365/375 The likelihood ratio is calculated using LR+ = sensitivity / (1- specificity). CONCLUDING: Hence, to find the likelihood ratio that is associated with a serum biomarker concentration greater than 100 U/mL, we will perform the calculations as outlined in the clinical reasoning step. RESPONSE: Let's perform the calculations. First, calculate sensitivity and specificity. Sensitivity = 120/336 ≈ 0.357 Specificity = 365/375 ≈ 0.973 Next, calculate LR+ = sensitivity/(1- specificity) which is 0.357/ (1- 0.973) ≈ 13. So, (C) 13 is the correct answer and gives us the likelihood ratio associated with a serum biomarker concentration greater than 100 U/mL in this HIV diagnostic test.

**AI_B:** RESTATING THE QUESTION: The question asks for the likelihood ratio associated with a serum biomarker concentration of greater than 100 U/mL, in a newly developed diagnostic test for HIV infection. The likelihood ratio is a method used in inferential statistics to compare the fit of different models to the observed data in a sample. In medicine, it is commonly used to evaluate the reliability of a diagnostic test in identifying a disease, like HIV in this case. It is calculated as the likelihood that a given test result would be expected in a patient with the target disorder compared to the likelihood that the same result would be expected in a patient without the target disorder. PLANNING: The likelihood ratio is defined as the probability of a positive test result in those with the disease (true positives), divided by the probability of a positive test result in those without the disease (false positives). The true positive rate, or sensitivity, is the number of patients with the condition who test positive divided by the total number with the condition. The false positive rate, or 1-specificity, is the number of patients without the condition who test positive divided by the total number without the condition. However, these assumptions are based on the quality and accuracy of the diagnostic test. CLINICAL REASONING: In this scenario, the summary statistics provided help compute the likelihood ratio for the serum biomarker concentration greater than 100 U/mL. From the information given, for a serum biomarker concentration of greater than 100 U/mL, the number of true positives is 120 (i.e., those with HIV who tested positive) and the number of false positives is 10 (those without HIV who tested positive). The total number of confirmed HIV cases is 336 and the total number of individuals without HIV is 375. CONCLUDING: The formula for the Positive Likelihood Ratio (LR+) = sensitivity / (1-specificity). Sensitivity (True Positive Rate) would be calculated as 120 / 336 = 0.357 and 1-Specificity (False Positive Rate) would be calculated as 10 / 375 = 0.027. By inserting the found values into the equation, the Positive Likelihood Ratio (LR+) = 0.357 / 0.027 ≈ 13.2, suggesting that it's more likely for individuals with a concentration of >100 U/mL to have HIV as compared to those who do not. RESPONSE: Thereby, the likelihood ratio associated with a serum biomarker concentration greater than 100 U/mL for those tested individuals would be closest to the provided option (C) 13.

**AI_C:** RESTATING THE QUESTION: The question is asking us to determine the likelihood ratio for the new diagnostic test for HIV infection given certain concentration levels of a serum biomarker. Importantly, we are focusing on concentrations greater than 100 U/mL. The likelihood ratio in this context is a statistical measure used in diagnostic testing that combines both the sensitivity and specificity of the test. Sensitivity is a measure of how well a test correctly identifies positive instances of a condition, while specificity indicates how well a test correctly identifies negative instances of a condition. PLANNING: To determine the likelihood ratio, we first need to calculate the sensitivity and specificity of the test for a serum biomarker concentration greater than 100 U/mL. However, there are assumptions involved in these calculations. The sensitivity of the test can be calculated using the formula: sensitivity = true positives / (true positives + false negatives). In this context, true positives are HIV positive individuals who tested positive with the serum biomarker concentration greater than 100 U/mL, and false negatives are HIV positive individuals who didn't reach this concentration level. Specificity, on the other hand, is calculated as: specificity = true negatives / (true negatives + false positives). True negatives are the HIV negative individuals who tested negative with the serum biomarker concentration greater than 100 U/mL, and false positives are HIV negative individuals that crossed this threshold. We need to calculate these parameters first in order to then calculate the likelihood ratio, which is expressed by the formula: likelihood ratio = sensitivity / (1 - specificity). CLINICAL REASONING: From the question, we know that there were 120 individuals who were positive for HIV and had a serum biomarker concentration greater than 100 U/mL. Since the total number of individuals who are positive for HIV is 336, we can calculate the sensitivity of the test as sensitivity = 120 / 336. For specificity, we know that there are 10 individuals who were negative for HIV but had a serum biomarker concentration greater than 100 U/mL (these are the false positives). Also, given that the total number of individuals that are negative for HIV is 375, the true negatives are 375 - 10, so we can calculate specificity as specificity = 365 / 375. CONCLUDING: After we have calculated sensitivity and specificity, we can compute the likelihood ratio as: likelihood ratio = sensitivity / (1 - specificity). This will give us the answer to the question. RESPONSE: Given the provided data, we first calculate sensitivity and specificity. Sensitivity = 120/336 = 0.357, and specificity = 365/375 = 0.973. Plugging these values into the formula for the likelihood ratio gives us: likelihood ratio = 0.357 / (1 - 0.973) = 13.3, closest to choice (C) 13.

**Facilitator** The question asks for the likelihood ratio associated with a serum biomarker concentration greater than 100 U/mL in a new diagnostic test for HIV infection. The likelihood ratio is a statistical measure used in diagnostic testing that combines both the sensitivity and specificity of the test. Sensitivity is a measure of how well a test correctly identifies positive instances of a condition, while specificity indicates how well a test correctly identifies negative instances of a condition. To calculate the likelihood ratio, we first need to calculate the sensitivity and specificity of the test for a serum biomarker concentration greater than 100 U/mL. The sensitivity of the test can be calculated using the formula: sensitivity = true positives / (true positives + false negatives). In this context, true positives are HIV positive individuals who tested positive with the serum biomarker concentration greater than 100 U/mL, and false negatives are HIV positive individuals who didn't reach this concentration level. Specificity, on the other hand, is calculated as: specificity = true negatives / (true negatives + false positives). True negatives are the HIV negative individuals who tested negative with the serum biomarker concentration greater than 100 U/mL, and false positives are HIV negative individuals that crossed this threshold. From the question, we know that there were 120 individuals who were positive for HIV and had a serum biomarker concentration greater than 100 U/mL. Since the total number of individuals who are positive for HIV is 336, we can calculate the sensitivity of the test as sensitivity = 120 / 336. For specificity, we know that there are 10 individuals who were negative for HIV but had a serum biomarker concentration greater than 100 U/mL (these are the false positives). Also, given that the total number of individuals that are negative for HIV is 375, the true negatives are 375 - 10, so we can calculate specificity as specificity = 365 / 375. After we have calculated sensitivity and specificity, we can compute the likelihood ratio as: likelihood ratio = sensitivity / (1 - specificity). Given the provided data, we first calculate sensitivity and specificity. Sensitivity = 120/336 = 0.357, and specificity = 365/375 = 0.973. Plugging these values into the formula for the likelihood ratio gives us: likelihood ratio = 0.357 / (1 - 0.973) = 13.3, closest to choice (C) 13. Therefore, the likelihood ratio that is associated with a serum biomarker concentration greater than 100 U/mL is (C) 13.
